# Positive narrative writing reduces music performance anxiety more effectively than traumatic narrative

**DOI:** 10.3389/fpsyg.2026.1846807

**Published:** 2026-09-16

**Authors:** Xiaofen Song

**Affiliations:** Music Education College, Wuhan Conservatory of Music, Wuhan, Hubei, China

**Keywords:** anxiety level regulation, core stressors, expressive writing intervention, music performance anxiety, positive narrative

## Abstract

Music performance anxiety (MPA) is a multidimensional construct that may impair performance quality and mental health, depending on individual and contextual factors. Current interventions are often costly, require specialized expertise, or lack feasibility. Expressive writing intervention (EWI) is low-cost and easy to implement. This study randomly assigned 120 music students with high MPA to three 3-day writing conditions: positive narrative (describing successful performances), traumatic narrative (describing stressful performances), or neutral control. MPA was measured pre- and post-intervention using the Kenny Music Performance Anxiety Inventory, and writing content was qualitatively analyzed. Results showed that positive narrative significantly reduced MPA (mean decrease = 35.27, *p* < 0.01), traumatic narrative also reduced MPA but less so (mean decrease = 21.55, *p* < 0.01), while the control group showed no change (*p* > 0.05). Qualitative analysis identified personal, repertoire, and situational stressors. These findings indicate that the content of expressive writing matters: positive narrative writing is more effective than traumatic narrative in alleviating MPA.

## 1 Introduction

Music Performance Anxiety (MPA) is a pervasive challenge in a musician's career, potentially harming mental health (Irie et al., 2023; Spahn et al., 2021, 2023) and significantly impacting performance quality (Sokoli et al., 2022; Passarotto et al., 2023; Jha et al., 2022). Recent reconceptualizations have challenged the medicalized framing of MPA, arguing that it should be viewed not as a disorder but as a normal and adaptive response to the pressures of performance (Herman and Clark, 2023). Research indicates that performers' MPA symptoms can manifest as physiological reactions like increased heart rate and trembling hands. These symptoms can appear during the preparation phase and persist until the performance ends, with physiological reactions peaking just before going on stage. Studies also show that performers may experience MPA behavioral symptoms like distraction during the performance, leading to mistakes and affecting quality, with symptoms gradually subsiding after the performance (Irie et al., 2023; Spahn et al., 2023). Further research points to individual differences in MPA symptoms, classifying MPA into three types: low-symptom, high-efficacy type; type that alleviates during performance; and type that continuously worsens (Kenny, 2011; Ryan et al., 2023). Factors such as musical background, student age, self-esteem, and practice time are positively correlated with MPA (Ryan et al., 2023).

Regarding MPA coping strategies, one study focused on piano learners from the perspective of self-regulated learning (SRL), which refers to learners' ability to manage their own cognitive, motivational, and emotional processes during learning. Using questionnaires and interviews, it revealed that SRL enhances self-efficacy and alleviates MPA through after-class reflection, confirming a positive correlation between self-efficacy and academic performance, and a negative correlation between anxiety and performance (Dong and Gedviliene, 2025). Another study, targeting university music majors, explored MPA from a visual rehearsal perspective, which involves mentally simulating the performance environment and stage scenarios to reduce anxiety. Through interviews and performance diary analysis, it revealed that students use visual rehearsal to familiarize themselves with the stage environment and get accustomed to the presence of an audience to alleviate MPA. The study also found that pre-performance psychological preparation and visual rehearsal are core strategies, while during the performance they combine breathing relaxation and self-talk for anxiety management (Huang and Song, 2021). These strategies, while effective, often require ongoing effort and practice; in contrast, expressive writing is a brief, one-time, low-cost intervention that can be easily implemented before a performance.

Expressive Writing Intervention (EWI) is a method of self-regulating psychological state through written language. It involves individuals expressing their inner emotions, thoughts, and experiences in written form to alleviate psychological stress and promote mental health. Depending on the writing style and requirements, expressive writing can be divided into free and structured types: free writing encourages individuals to freely narrate unpleasant experiences and describe feelings, while structured writing requires a structured description according to a fixed format, with guiding instructions set at different stages (Guo et al., 2017; Frattaroli, 2006). Currently, EWI has demonstrated positive mental health promotion effects across various groups, including the general population, patients with post-traumatic stress disorder, and athletes (Frattaroli, 2006; Liu et al., 2023; Yang and Li, 2020; Mankad et al., 2009; Hudson and Day, 2012). Particularly in sports, it is used to improve the mental health of injured athletes, alleviate emotional issues, and enhance confidence, while also being used for ordinary athletes to relieve stress, and its benefits have been extended to university students (Batista et al., 2022). A systematic review of psychological interventions for music performance enhancement identified expressive writing as one of the few interventions with consistent positive effects on expert-rated performance quality (Lubert and Gröpel, 2024), supporting its potential as a practical self-regulation tool for musicians.

The psychological mechanisms of expressive writing and MPA are highly compatible. Existing research shows that the core psychological characteristics of MPA manifest as excessive worry about mistakes fear of negative evaluation, and post-performance rumination and self-criticism about errors (Irie et al., 2023). This persistent “negative rumination” exacerbates the anxiety experience.

A useful theoretical framework for understanding the differential effects of writing content is Gross (2015) process model of emotion regulation, which distinguishes between different strategies for managing emotional responses. Among these strategies, cognitive reappraisal—the reinterpretation of a situation to alter its emotional impact—has been shown to be particularly effective in reducing anxiety (Gross, 2015). In the context of MPA, positive narrative writing may function as a form of reappraisal, prompting performers to reinterpret performance situations as opportunities rather than threats. In contrast, traumatic narrative writing may reinforce negative rumination, thereby maintaining or exacerbating anxiety (Twitchell et al., 2025). This framework suggests that it is not simply the act of writing that matters, but the type of cognitive process it activates.

This theoretical perspective aligns with research distinguishing between facilitative and debilitative arousal in MPA. The perception of arousal symptoms plays a critical role in determining their impact: facilitative arousal occurs when symptoms such as increased heart rate and trembling are interpreted as positive excitement that enhances performance, whereas debilitative arousal occurs when the same symptoms are perceived as threatening and impair performance (Murphy et al., 2024; Osborne et al., 2020).

Their study provides direct evidence for this mechanism: expressive writing significantly reduced anxiety levels in piano students, decreased self-critical dialogue, and reduced performance errors by approximately 50% in highly anxious students (Tang and Ryan, 2020). Recent studies have begun to explore the effects of combining different writing paradigms (Batista et al., 2022). However, existing research has largely focused on overall intervention effects, and the differential impact of different writing themes (e.g., traumatic narrative, positive narrative) on MPA remains under explored.

Therefore, this study aims to compare the differential intervention effects of different writing themes (positive narrative, traumatic narrative, neutral writing) on MPA, providing empirical evidence for optimizing EWI protocols. To this end, the study selected 120 music major students with relatively high MPA from a music academy in China and conducted a 3-day EWI experiment. Combining pre- and post-MPA measurements with text content analysis, and by comparing the intervention effects of different writing themes, this study clarifies the differential role of writing content in alleviating MPA, providing a basis for designing personalized writing protocols. By comparing the effect differences of different writing themes, this study confirms that the type of writing theme directly affects MPA intervention outcomes, clarifying that “what to write” is more important than “whether to write,” thus providing a basis for designing personalized writing protocols.

## 2 Materials and methods

### 2.1 Research participants

This study targeted 120 university students majoring in music performance at a professional music academy. All participants were screened using three subscales of the Kenny Music Performance Anxiety Inventory (K-MPAI; Kenny, 2011): Proximal Somatic Anxiety and Worry About Performance (11 items), Worry/Dread Focused on Self/Other Scrutiny (8 items), and Memory (2 items), totaling 21 items. A total score ≥ 60 was required for inclusion and public performance experience in the past two years (defined as at least 5 performances per year). This threshold is consistent with the typical performance frequency of music majors in Chinese conservatories, where students typically perform in 3–5 public concerts or exams annually (e.g., end-of-semester juries, studio classes, and departmental recitals). This threshold was chosen based on previous research distinguishing regular performers from occasional ones (e.g., Ryan et al., 2023 used a similar cutoff). Individuals currently receiving psychological treatment or taking anti-anxiety medication were excluded. The final sample had an average age of 19.6 years (SD = 1.3), covering piano (38), vocal (32), cello (25), flute (15), and other instruments (10), forming a cross-specialty, representative group for anxiety intervention research. This study was approved by the Clinical Research Ethics Committee of Renmin Hospital of Wuhan University (Ethics Approval Number: WDRY2024-K112). All participants voluntarily signed informed consent forms and had the right to withdraw at any time. For potential negative emotions induced in the traumatic narrative group, the study provided necessary guidance and informed them of psychological support resources. All data were anonymized and kept strictly confidential.

### 2.2 Experimental design

This study employed an exploratory sequential design within a mixed-methods approach. In December 2024, a structured writing intervention experiment was conducted over 3 days, which follows the standard expressive writing protocol (Pennebaker and Beall, 1986), with 120 music performance majors. The quantitative part of this study is a randomized controlled trial (RCT). Participants were randomly assigned to three groups using a computer-generated random number sequence (Excel RAND function) and engaged in recording performance-related events for 20 minutes daily over three consecutive days. The traumatic narrative group focused on participants' previous unpleasant music performance experiences (e.g., major mistake events). The positive narrative group described successful performance experiences of equivalent length and summarized personal strengths. The control group wrote on neutral topics. All students completed a post-test within 24 h after the intervention for quantitative assessment of anxiety level changes.

The positive narrative group sequentially completed tasks involving scenario descriptions of successful performance experiences, exploration of positive coping strategies, and formulation of future performance goals. The traumatic narrative group detailed challenging performance events, reflected on the process of handling mistakes, and summarized strategies for transforming pressure. The control group primarily objectively recorded music-related activities from the past week, choosing one event per day for factual description, maintaining objectivity without involving thoughts and feelings. Guiding instructions for different writing contents are shown in Tables 1–3.

**Table 1:** Positive narrative group writing protocol.

Session	Theme	Guiding instructions
1	Detailed description of a successful performance	Please recall and describe a music performance experience that made you feel proud and satisfied. Describe the performance scene, stage setup, audience reaction, your role and position, and the cause, process, and outcome of the performance in detail. Try to vividly recreate the situation and your emotional experience, including your preparation before the performance, your feelings during the performance, and your reaction after the performance.
2	Exploration of positive coping strategies	Reflect on the successful music performance you described yesterday and write about the following: 1. What specific actions did you take to ensure the success of this performance during your preparation? 2. When you encountered minor challenges or nervousness during the performance, what methods did you use to adjust your state? 3. Describe a time before a performance when you felt particularly nervous but ultimately succeeded in overcoming it, and how you did so.
3	Positive Significance and Future Outlook	Revisit the music performance experience you described earlier and answer the following questions: 1. What positive significance does this successful performance experience have for your personal growth and musical development? 2. From this experience, what skills or mental adjustment methods have you learned that can be applied to future performances? 3. Based on this experience, set a specific small goal and describe how you will apply these learned lessons in your next performance.

**Table 2:** Traumatic narrative group writing protocol.

Session	Theme	Guiding instructions
1	Detailed description of a challenging performance	Please recall and describe a music performance experience that made you feel confused, upset, or uncomfortable. Describe the performance scene, stage setup, audience reaction, your role and position, and the cause, process, and outcome of the performance in detail. Express your emotions and feelings authentically, including your nervousness before the performance, mistakes or difficulties during the performance, and your reflection after the performance.
2	Coping strategies and reflection	Reflect on the challenging music performance you described yesterday and write about the following: 1. How did you initially cope with feelings of nervousness or anxiety during this performance? 2. When things did not go as planned, what measures did you take to minimize the negative impact? 3. Describe a time during a performance when you made a mistake and how you adjusted your mindset to continue performing.
3	Growth and Advice	Revisit the music performance experience you described earlier and answer the following questions: 1. From this experience, what did you learn about your ways of coping with stress and anxiety? 2. Based on this experience, what preparations do you think you could make in advance to better handle similar situations in the future? 3. If a fellow student were to encounter a similar situation, based on your experience, what advice would you give them to help them better deal with pressure and challenges in performance?

**Table 3:** Control group writing protocol.

Session	Theme	Guiding instructions
1	Listing and brief description of events	Please list and briefly describe 5 events related to music performance from the past week. These events could be related to daily practice, rehearsals, performance preparation, or other music-related activities.
2	Description of the second event	From the remaining 4 events, choose one and describe it in detail.
3	Description of the third event	From the remaining 3 events, choose the last one and describe it in detail.

### 2.3 Interview design

Using participant codes to match pre- and post-test data, 10 students with the largest MPA reductions were purposively selected for follow-up interviews to explore potential mechanisms. The sample size was determined by thematic saturation, as no new themes emerged after the eighth interview (see Table 4 for basic information on the students). The following questions were used to further explain the pathway of EWI's effect on MPA: “Please describe the most impressive performance experience you have had.” “What new understandings about your coping strategies for MPA did you gain through the writing tasks?” “Do you think the writing tasks helped you better understand the source of your performance anxiety?” “How do you evaluate the impact of the writing intervention on your psychological state? (e.g., reducing anxiety, enhancing confidence, etc.)”

**Table 4:** Basic information of interview participants.

Student	Major	2-year performance experience (times/year)	MPA pre-test score	MPA post-test score
Student 1	Pipa	5	203	109
Student 2	Vocal	6	230	112
Student 3	Flute	5	220	116
Student 4	Piano	8	190	106
Student 5	Guitar	4	195	131
Student 6	Guzheng	5	220	94
Student 7	Trumpet	3	234	110
Student 8	Piano	4	189	112
Student 9	Trombone	3	177	157
Student 10	Piano	5	174	102

### 2.4 Data analysis

The qualitative analysis was guided by the first author (X. Song). This study first used SPSS 25 software to conduct variance analysis on the anxiety levels of different intervention groups to determine whether there were significant differences in the impact of different intervention methods on anxiety levels. To deeply analyze the causes of anxiety, the study further used NVivo 14 software for qualitative analysis of the text materials from the free writing group and the positive writing group. Through three-level coding of the writing content, core stressors of MPA (e.g., “doubt about ability,” “perfectionism”) were extracted from anxiety-related descriptions in the original statements (e.g., “fingers trembling,” “fear of being laughed at”) to gain an in-depth understanding of the main causes of MPA.

To establish inter-rater reliability, a second coder coded 20% of the interview transcripts (*n* = 2 participants, 33 coding units) using the same coding framework (personal factors, repertoire-related pressure, performance context pressure, and EWI intervention effects). Cohen's kappa coefficient was calculated to assess coding agreement. The analysis yielded a kappa value of 0.739, indicating substantial agreement between coders (Landis and Koch, 1977). The six coding discrepancies were discussed and resolved through consensus.

## 3 Results

### 3.1 Intervention effects of positive and traumatic narratives on MPA

To ensure the rigor of the grouping, this study conducted a variance analysis of MPA among students in different writing groups before the intervention. Results, shown in Table 5, indicated no significant difference in MPA among the three groups before the intervention (*p* > 0.05).The small standard deviations in pre-test scores (Table 5) reflect the homogeneity of the sample. All participants were recruited from the same conservatory, and the inclusion criterion of K-MPAI ≥ 60 further ensured that all had similarly high levels of baseline MPA. The selected K-MPAI subscales demonstrated excellent internal consistency in this sample (Cronbach's α = 0.951).

**Table 5:** ANOVA of pre-intervention MPA across different writing groups.

ANOVA results (pre-intervention)						
Analysis item	Group	*N*	Mean	SD	*F*	*p*
Group	Traumatic narrative	40	202.48	1.96	1.944	0.148
	Control	40	202.63	2.01		
	Positive narrative	39	201.79	2.03		
	Total	119	202.31	2.01		

^*^p < 0.05, ^**^p < 0.01.

During the intervention implementation, one participant in the positive narrative group missed the first day's writing task but completed all writing content on the second day. Therefore, this participant was included in the final analysis, resulting in a sample size of 40 for this group. After three days of EWI, the three groups showed significant differences in MPA levels (*F* = 361.352, *p* < 0.01), as shown in Table 6. The effect size for this ANOVA was very large (η^2^ = 0.861).

**Table 6:** ANOVA of post-intervention MPA across different writing groups.

Analysis item	Group	*N*	Mean	SD	*F*	*p*
Group	Traumatic narrative	40	180.93	5.85	361.352	0.000^**^
	Control	40	202.71	1.8		
	Positive narrative	40	166.46	8.55		
	Total	120	183.36	16.13		

^*^p < 0.05, ^**^p < 0.01.

For the positive narrative group, the intervention produced a very large pre-post effect (Cohen‘s *d* = 4.01). Table 7 further analyzes the changes in MPA levels before and after the intervention. The positive narrative group showed a mean MPA decrease of 35.27 (*p* < 0.01) after the intervention, the traumatic narrative group showed a mean MPA decrease of 21.55 (*p* < 0.01), while the control group showed no significant change (*p* > 0.05). This indicates that both positive and traumatic narratives had significant effects on MPA, with the positive narrative having a stronger alleviating effect. *Post-hoc* comparison showed that the positive narrative group had significantly lower MPA than the traumatic narrative group (mean difference = 14.47, *p* < 0.01), with a large effect size (Cohen's *d* = 1.98).

**Table 7:** Paired *t*-test analysis of MPA levels pre- and post-intervention.

Analysis item	Group	*N*	Mean	SD	Mean difference	95% CI of difference	*T*	*F*	*p*
Positive narrative	Pre-test	40	201.73	2.04	35.27	32.472–38.078	25.377	43.433	0.000^**^
	Post-test	40	166.46	8.55					
	Total	80	184.09	18.79					
Traumatic narrative	Pre-test	40	202.48	1.96	21.55	19.592–23.518	22.08	47.638	0.000^**^
	Post-test	40	180.93	5.85					
	Total	80	191.7	11.68					
Control	Pre-test	40	202.63	2.01	−0.08	−0.934–0.764	−0.199	78	0.842
	Post-test	40	202.71	1.8					
	Total	80	202.67	1.89					

^*^p < 0.05, ^**^p < 0.01.

### 3.2 Identification of core stressors

This study further analyzed the emotional vocabulary in the three-day writing content of 120 participants to identify the main stressors of MPA. First, concept construction was performed on the expressive writing texts of the 120 participants, extracting 38 concept words and phrases related to performance stressors (e.g., “traumatic memory of performance failure,” “audience expectations,” “perfectionism”). Following concept construction, 10 secondary themes were extracted, which were ultimately summarized into three core stressors: personal factor stressors, repertoire factor stressors, and performance situation factor stressors (Table 8).

**Table 8:** Coding results for main sources of stress in music performers.

Level 1 coding (frequency)	Level 2 coding	Level 3 coding
Personal factor stressors (32)	Confidence and self-efficacy	Self-evaluation
		Feedback from others
		Successful experiences
		Sensitivity to audience evaluation
		Team responsibility
	Personality traits and coping styles	Introversion/extraversion
		Perfectionism
		Ways of coping with stress
	Anxiety history and psychological resilience	Past anxiety experiences
		Changes in anxiety levels
		Psychological resilience
		Traumatic memories of performance failure
		Insufficient proactive regulation ability
	Physiological state and physical reactions	Finger stiffness/pain
		Muscle tension
		Increased heart rate
		Shortness of breath
Repertoire factor stressors (25)	Difficulty and technical challenges	Technical difficulty
	Musical complexity
	Familiarity with the piece
	Practice level and preparation adequacy	Practice time
		Practice effectiveness
		Adequacy of preparation
	Performance technique and stage presence	Playing technique
		Stage presence
		Body language and eye contact
Performance situation factor stressors (29)	Audience pressure and expectations	Audience size
	Audience type
	Audience expectations
	Venue familiarity and adaptability	Familiarity with venue
		Adaptability to venue
		Venue atmosphere
		Equipment noise
		Visual distractions
	Importance and consequences of performance	Performance purpose
		Consequences of performance
		Source of pressure

### 3.3 Pathways of expressive writing's effect on music performance anxiety: preliminary findings based on interviews

To preliminarily explore the mechanism of expressive writing's effect on MPA, this study conducted semi-structured interviews with 10 students who showed significant intervention effects. The interview results suggested that expressive writing may impact MPA through the following pathways. It should be noted that these pathways are still preliminary hypotheses and need to be verified by future research, such as through quantitative text analysis. The word cloud is shown in Figure 1.

**Figure 1:**
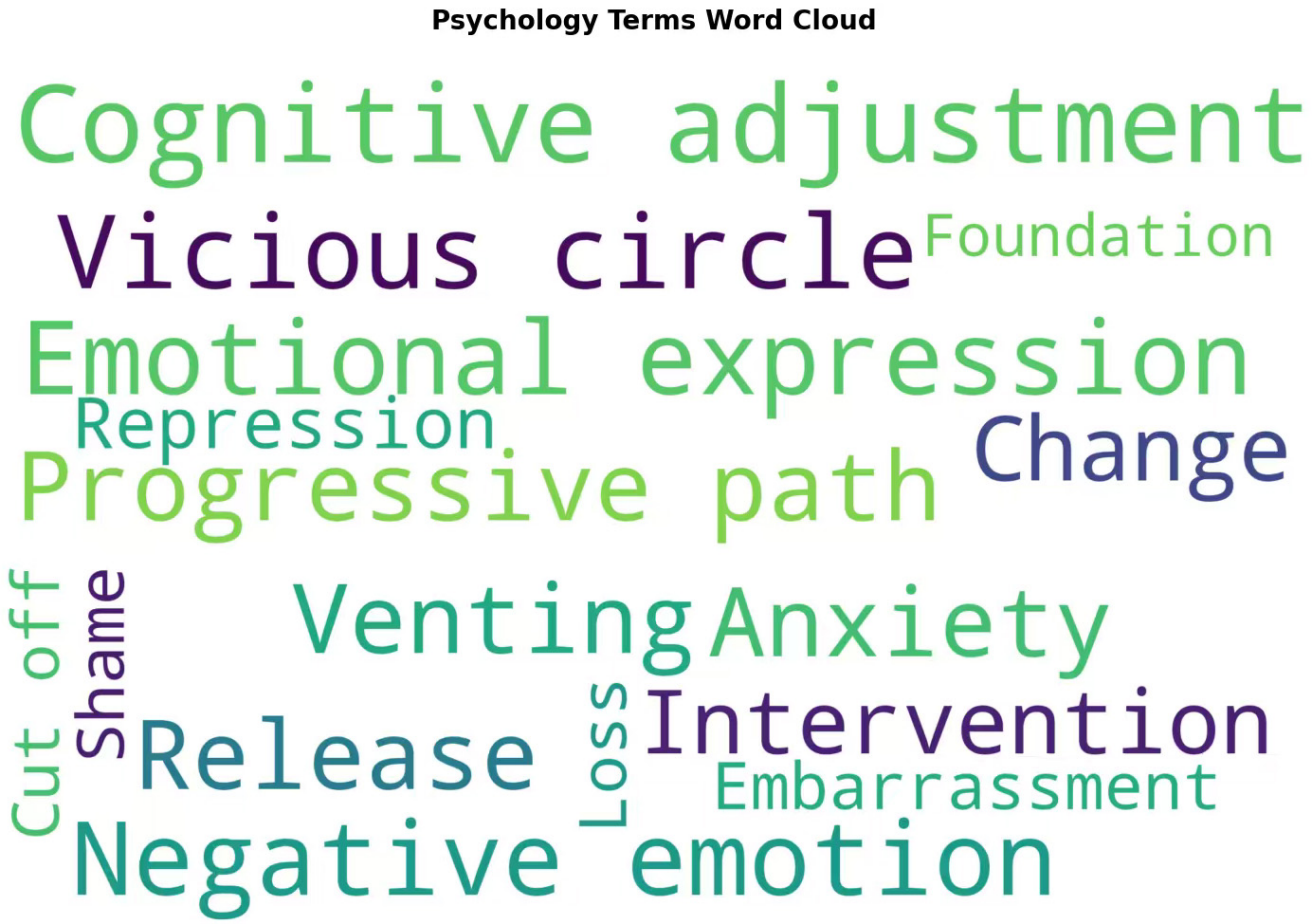
Intervention effect of writing on emotional release.

### 3.4 Emotional expression and catharsis

In the interviews, several students mentioned that writing helped them confront and release negative emotions brought on by performance anxiety. For example, Student 1, when describing a Pipa performance mistake during a sophomore welcome party, wrote: “The moment the string broke, I seemed to hear the sighs from the audience, and the embarrassment and shame washed over me like a tide.” Through writing, he re-experienced this emotion but also realized: “This emotion is not a flood beast; it is the driving force for my progress.” Student 5, describing a guitar performance mistake during a freshman welcome party, also mentioned: “Writing gave me the chance to pour out that feeling of loss and frustration instead of suppressing it.” This expression and catharsis of emotions helped students break the vicious cycle of anxiety suppression, laying the foundation for subsequent cognitive adjustment.

### 3.5 Cognitive adjustment

Interview results indicated that in many performance situations, performers often focus excessively on minor flaws, leading to negative cognition, continuously generating negative emotions, and interfering with subsequent performance states. Cognitive restructuring can guide performers to reflect on past positive and successful performance experiences through writing, promoting positive thinking, effectively reducing the probability of MPA occurrence, and enhancing the performance experience. As Student 4 reflected on a high school piano competition mistake: “I was overly pursuing perfection, which led to a mistake on the ornament. But writing made me realize that music is not about competition, but about conveying emotion.” This suggests that writing prompts performers to re-evaluate performance experiences, correct negative perceptions of stressors, break the vicious cycle, and shift cognitive focus from over-avoidance of errors to optimizing performance through dimensions like emotional expression and creative engagement.

### 3.6 Focus on coping strategies

When performers shift their thinking in writing from negative cognitions like anxiety and mistakes to optimizing performance preparation details, they can both alleviate MPA and actively address problems with concrete actions, reducing the likelihood of anxiety occurrence and enhancing a sense of control. When writing content shifts the performer's reflection focus from the “mistake” itself to “how to optimize,” it not only reduces interference from performance anxiety but also, through “problem concretization,” forms coping patterns adapted to performance situations, thereby constructing a more proactive performance psychology framework. Student 6, describing an incident where a Guzheng nail came loose during a Junior New Year's Eve gala, mentioned: “Writing made me realize that accidents are inevitable, but preparation can be more thorough.” Subsequently, she realized the importance of “double-securing the nails with tape before every performance and preparing a spare nail kit.” Through reflection after writing, she developed a more comprehensive preparation strategy. This shows that writing prompts them to sort out problems and subsequently adjust their behavior.

## 4 Discussion

### 4.1 Main stressors revealed by EWI

Through qualitative analysis of participants‘ writing texts, this study revealed various stressors of MPA. At the personal level, a lack of confidence and self-efficacy was a common issue reported by participants. They often doubted their abilities, experiencing self-doubt stemming from self-evaluation, feedback from others, and a lack of confidence from successful experiences. This lack of confidence further led to heightened sensitivity in front of an audience, fear of negative evaluation, and thus exacerbated anxiety. Liu (2021) also pointed out that evaluation threat is one of the core causes of MPA, where performers fall into an anxiety cycle due to excessive concern about others' evaluations. Zhang (2024) also indicated that intrinsic motivation in music performance is the most critical factor influencing MPA, while personality traits like conscientiousness and emotional stability are negatively correlated with anxiety.

Furthermore, this study found that personality traits and coping styles significantly influence MPA. Zheng and Chen (2024) also proposed that personality traits play a key role in the formation of MPA, with individuals high in neuroticism, perfectionism, introversion, low self-efficacy, high sensitivity, and low agreeableness being more susceptible to anxiety. Multiple participants in this study exhibited perfectionistic tendencies, noting that their excessive pursuit of flawlessness led to self-blame and anxiety upon making mistakes. Simultaneously, different coping styles when facing pressure contributed to varying anxiety levels among participants. Some participants struggled to effectively alleviate anxiety due to inappropriate coping methods or insufficient proactive regulation ability. Anxiety history and psychological resilience were also important personal factors. Past anxiety experiences profoundly impacted participants, shaping different levels of psychological resilience. Some participants mentioned traumatic memories of performance failure, which became a significant source of their anxiety. Insufficient psychological resilience made it difficult for them to recover from anxiety. Performers with a history of anxiety and low psychological resilience are susceptible to the influence of past anxiety. Additionally, when repertoire difficulty exceeds their technical ability, audience expectations are high, the venue is unfamiliar, or the performance holds significant consequences, performers who focus heavily on technique, evaluation, have poor adaptability, or value outcomes highly are also prone to anxiety from these stressors.

The identification of three distinct MPA trajectories (low-symptom high-efficacy, performance-alleviated, and continuously worsening) highlights the heterogeneity of performers'experiences. Participants with perfectionistic tendencies or low psychological resilience were more likely to experience persistent anxiety, consistent with prior findings (Zheng and Chen, 2024). This variability suggests that personalized writing interventions—matching narrative type to individual disposition—may enhance efficacy. For example, highly self-critical individuals might benefit more from positive narrative, whereas those who already use adaptive coping may require different content. Future research should examine individual moderators such as trait rumination or self-efficacy to optimize intervention matching. This individualized approach is consistent with the call to move beyond a one-size-fits-all, pathology-focused model of MPA and instead recognize the diversity of performers' experiences (Herman and Clark, 2023).

### 4.2 Differential effects and mechanisms of positive and traumatic narratives on MPA

This study found that positive narrative had a more pronounced alleviating effect on MPA than traumatic narrative. This might be because positive narrative significantly increased the use of cognitive process words (e.g., “understand,” “accept”), prompting writers to actively explore positive coping strategies and growth meanings within the event, effectively enhancing positive emotions and reducing negative emotions (Jiang, 2022). This finding aligns with Gross (2015) process model of emotion regulation, specifically the strategy of cognitive change (reappraisal),

This finding is also consistent with Twitchell et al. (2025), who argued that the key factor in whether MPA becomes helpful or harmful lies in the appraisal of the stressor rather than the stressor itself. In our study, participants in the positive narrative group were prompted to reinterpret their performance experiences—shifting from threat-focused to challenge-focused appraisals—whereas those in the traumatic narrative group tended to reinforce threat appraisals. This suggests that expressive writing does not merely change the content of performers' thoughts, but fundamentally alters their appraisal processes, which in turn shape the emotional and physiological experience of MPA. These findings corroborate the broader theoretical claim that situational factors do not determine stress responses directly; rather, it is the individual's appraisal of those situations that matters most (Gross, 2015; Twitchell et al., 2025).

Positive narrative writing prompts individuals to reappraise their performance experiences, shifting focus from potential failures to strengths and growth. In contrast, traumatic narrative may inadvertently reinforce maladaptive rumination, a form of perseverative cognition that maintains anxiety. Although traumatic narrative also releases emotions, its focus on negative events offers limited benefits for psychological growth and the enhancement of positive emotions, hence its weaker effect on alleviating MPA. Regarding potential mechanisms, the interview results suggested that expressive writing may positively impact performance anxiety through pathways such as emotional catharsis and cognitive restructuring. This speculation aligns with Jiang (2022) findings that expressive writing promotes emotional improvement through cognitive process words, but still requires verification using text analysis data (Qiu et al., 2022). Simultaneously, writing can prompt students to re-evaluate performance experiences, modify ingrained perceptions of stressors, and establish positive cognitive patterns. Based on writing reflection, students also focus on coping strategies to mitigate performance risks, thereby alleviating anxiety.

The integration of quantitative and qualitative findings further strengthens the validity of our conclusions. Quantitatively, positive narrative writing produced a significantly larger reduction in MPA (Cohen's d = 1.98) than traumatic narrative. Qualitatively, participants in the positive narrative group reported cognitive restructuring (e.g., “music is not about competition, but about conveying emotion”) and a shift from self-criticism to constructive reflection. In contrast, those in the traumatic narrative group often remained focused on negative emotions and performance failures. This convergence suggests that the mechanism of benefit is not simply emotional disclosure, but the construction of a coherent, positive performance narrative that fosters adaptive reappraisal. This interpretation aligns with Herman and Clark (2023) reconceptualization of MPA, which emphasizes that the key to managing MPA lies not in eliminating anxious symptoms but in changing performers' responses to them. Positive narrative writing appears to facilitate precisely this shift—from threat-focused to challenge-focused appraisal. Similarly, Lubert and Gröpel (2024) identified expressive writing as one of the most promising interventions for enhancing music performance quality through self-regulation, further supporting the practical relevance of the present findings.

## 5 Limitations and future directions

This study has certain limitations as it falls within the scope of exploratory research and the results should therefore be interpreted cautiously. Its conclusions need to be verified by future experimental studies with larger samples and longer cycles. Firstly, the intervention period was only 3 days. The primary goal of this study was to examine the immediate effects of expressive writing on MPA, following the classic EWI paradigm (Pennebaker and Beall, 1986), which typically measures outcomes immediately post-intervention. Therefore, no follow-up measurements were included. The decision was also influenced by the exploratory nature of this study and resource constraints. However, the durability of the effect over 3–6 months remains unknown, and future studies should incorporate longitudinal designs to assess long-term stability. Although significant intervention effects were observed, it cannot differentiate between short-term emotional release effects and long-term stable anxiety alleviation. This limitation restricts the generalizability of the study's conclusions. Future research should extend the intervention period and include follow-up measurements to examine the duration and stability of intervention effects. Secondly, this study only compared three writing themes. Future research could explore the intervention effects of other writing themes on music performance anxiety. Finally, although the study observed individual differences in the application of writing, it did not delve into the specific manifestations and influencing factors of these individual differences. Additionally, this study used selected subscales from the K-MPAI rather than the full 40-item scale. Although the selected subscales showed high internal consistency (α = 0.951), future research may benefit from using the full scale for more comprehensive measurement.

## 6 Conclusion

Through a 3-day EWI experiment, this study delved into the intervention effects of different writing narrative paradigms (positive narrative vs. traumatic narrative) on MPA. The results indicated that positive narrative intervention significantly reduced anxiety levels in music performers, while traumatic narrative intervention also alleviated anxiety to some extent, but its effect was weaker than that of positive narrative. This study further revealed the structure of core stressors for MPA, encompassing dimensions such as personal factors, repertoire, and performance situations, providing a theoretical basis for subsequent precise interventions. Interview results preliminarily suggested that expressive writing may alleviate MPA through pathways like emotional expression, cognitive adjustment, and focusing on coping strategies. The reliability of these pathways requires verification through methods such as quantitative text analysis in future research.

This study confirms the effectiveness of EWI as a low-cost, easy-to-administer psychological regulation tool. This method does not require specialized venues or the involvement of a psychologist. Through structured writing exercises over 3–5 consecutive days, it can help music performers build a “readily accessible” anxiety buffer mechanism. This universality offers an innovative solution for the current shortage of psychological counseling resources in music academies, while also providing a replicable practical approach for self-management of performance anxiety.

This study provides new ideas and methods for alleviating MPA. Future research can expand and deepen this work in several aspects. First, the sample size can be expanded to include music performers from more diverse backgrounds and levels to enhance the generalizability and reliability of the findings. Second, the intervention duration can be extended to more comprehensively evaluate the long-term effects of expressive writing and its impact on the professional development of music performers. Furthermore, future research could explore the intervention effects of other writing themes, such as mixed themes or personalized themes, to provide richer and more precise writing intervention protocols. Simultaneously, an in-depth analysis of the impact of individual differences on writing intervention effects can be conducted to offer more personalized writing intervention plans for music performers. Finally, strengthening interdisciplinary collaboration and communication among psychology, musicology, and education can help explore solutions for MPA, providing more comprehensive support for the professional development and mental health of music performers.

## Data Availability

The raw data supporting the conclusions of this article will be made available by the authors, without undue reservation.
